# Plasma glucose kinetics and response of insulin and GIP following a cereal breakfast in female subjects: effect of starch digestibility

**DOI:** 10.1038/ejcn.2015.50

**Published:** 2015-04-08

**Authors:** F Péronnet, A Meynier, V Sauvinet, S Normand, E Bourdon, D Mignault, D H St-Pierre, M Laville, R Rabasa-Lhoret, S Vinoy

**Affiliations:** 1 Department de Kinésiologie, Université de Montréal, Montreal, Quebec, Canada; 2 Département de Nutrition, Mondelēz International R&D, Saclay, France; 3 Centre de Recherche en Nutrition Humaine Rhône-Alpes and Centre Européen de Nutrition pour la Santé, Lyon 1, Lyon, France; 4 Platform for Research in Obesity, Metabolism and Diabetes, Institut de Recherches Cliniques de Montréal and Département de nutrition, Faculté de médecine, Université de Montréal, Montreal, Quebec, Canada; 5 Département de Kinanthropologie, UQAM, Montreal, Quebec, Canada

## Abstract

**Background/Objectives:**

Foods with high contents of slowly digestible starch (SDS) elicit lower glycemic responses than foods with low contents of SDS but there has been debate on the underlying changes in plasma glucose kinetics, that is, respective contributions of the increase in the rates of appearance and disappearance of plasma glucose (RaT and RdT), and of the increase in the rate of appearance of exogenous glucose (RaE) and decrease in endogenous glucose production (EGP).

**Subjects/Methods:**

Sixteen young healthy females ingested in random order four types of breakfasts: an extruded cereal (0.3% SDS: Lo-SDS breakfast) or one of three biscuits (39–45% SDS: Hi-SDS breakfasts). The flour in the cereal products was labeled with ^13^C, and plasma glucose kinetics were measured using [6,6-^2^H_2_]glucose infusion, along with the response of plasma glucose, insulin and glucose-dependent insulinotropic peptide (GIP) concentrations.

**Results:**

When compared with the Lo-SDS breakfast, after the three Hi-SDS breakfasts, excursions in plasma glucose, the response of RaE, RaT and RdT, and the reduction in EGP were significantly lower (*P*<0.05). The amount of exogenous glucose absorbed over the 4.5-h postprandial period was also significantly lower by ~31% (*P*<0.001). These differences were associated with lower responses of GIP and insulin concentrations.

**Conclusions:**

Substituting extruded cereals with biscuits slows down the availability of glucose from the breakfast and its appearance in peripheral circulation, blunts the changes in plasma glucose kinetics and homeostasis, reduces excursions in plasma glucose, and possibly distributes the glucose ingested over a longer period following the meal.

## Introduction

Diets with a low glycemic index (GI), which are associated with a wide range of health benefits,^1, 2, 3^ are promoted as part of healthy nutritional habits.^4, 5^ However, many carbohydrate (CHO)-rich foods undergo processings that increase the digestibility of starch^6^ and thus have a high GI (>70).^1^ For this reason, there is a growing interest in developing low GI foods, particularly for breakfast.^6^

For similar content in macronutrients and fibers, the GI of starchy foods can be reduced by selecting starches with a higher amylose/amylopectin ratio^7^ and/or by using processes that preserve the structure of the grain and the crystalline structure of starch.^7, 8, 9^ The purpose of the present experiment was to compare the glycemic and hormonal response and plasma glucose kinetics following four breakfasts with a similar nutrient composition and containing a cereal product derived from the same starch but with different slowly digestible starch (SDS) content, that is, an extruded cereal containing 0.3% SDS (Lo-SDS breakfast) vs three slightly different biscuits obtained by a process that preserves starch resistance to digestion (Hi-SDS breakfasts: 39–45% SDS) (Table 1). Several studies have shown that differences in GI might not only reflect the differences in the rate of exogenous glucose appearance (RaE), but also the differences in endogenous glucose production (EGP), in the rate of total glucose appearance (RaT),^10, 11, 12, 13^ and in the glucose clearance rate.^12^ To compare plasma glucose kinetics following the two types of breakfast, the flour used in manufacturing the cereal products was intrinsically labeled with ^13^C and plasma glucose kinetics was measured using dual-tracer methodology with [6,6-^2^H_2_]glucose infusion. Owing to the slower digestibility of starch in the biscuits, we hypothesized that the response of plasma glucose, insulin and glucose-dependent insulinotropic peptide (GIP), and changes in plasma glucose kinetics will be smaller with the Hi-SDS than with the Lo-SDS breakfast.

## Materials and methods

### Subjects

**Figure 1:**
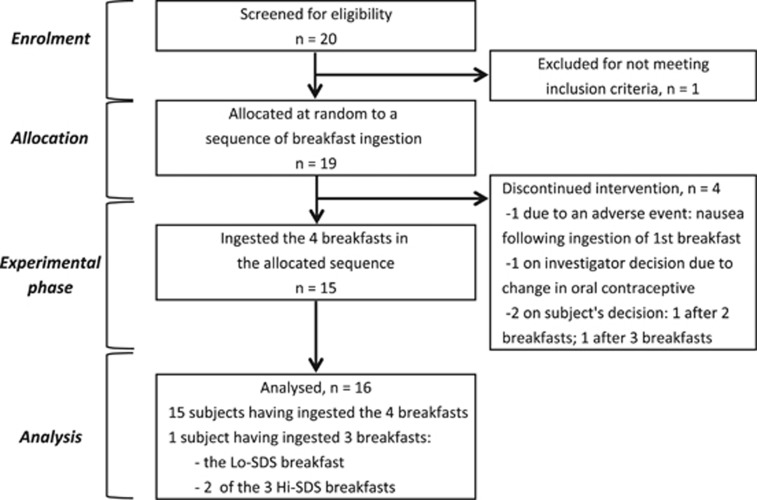
Flowchart of subject recruitment, group allocation, experimental phase and analysis.

This monocenter, randomized, balanced, open-label, crossover study with four products ingested by each subject in a random order was conducted on female subjects (18–40 years old, taking an oral contraceptive and moderately active (<3 h of exercise/week), body mass index between 20 and 25 kg/m^2^, stable body mass, non-smoker, moderate alcohol consumption (<20 g/day),^14^ under no medication, free of any health problem likely to interfere with the variables studied, as judged from medical history, clinical examination and biochemical and hematological analysis) (Figure 1). On the basis of a 3-day food diary at least 15% of the daily energy intake was provided at breakfast, and percentage energy intakes from CHO and fat were 50–55% and 30–35%, respectively, with the balance from protein.

The study was approved by the Faculty of Medicine Ethics Committee at University of Montreal and written informed consent was obtained from all subjects. On the basis of the previous results^15^ the expected difference in RaE between Hi- and Lo-SDS breakfasts was estimated to be 1 mg/kg/min. To detect this difference with a 70% power and a 5% α-risk, the number of subjects to be included was 14.^16^ Accounting for the balanced randomization block size, the sample size was set at 16.

### Cereal products and breakfasts

The cereal products were ingested as part of breakfasts that were isoenergetic and provided similar amounts of the three nutrients (Table 1). When compared with the extruded cereals, the three biscuits, produced using a rotary-moulding technology to limit starch gelatinization, had a very high content of SDS and lower GI as measured *in vivo* following WHO/FAO recommendations.^5, 17^ They slightly differed in their SDS to rapidly digestible starch and in their SDS to total starch ratios (0.63–0.81 and 0.42–0.46, respectively) as well as in their sugars to available CHO ratio (0.28–0.36) (Table 1).

The extruded cereals and biscuits were intrinsically enriched in ^13^C in the range of values of sugarcane used in manufacturing the cereal products (Table 1). For this purpose 0.2% of the flour used was obtained from soft wheat (*Triticum aestivum,* Scipion) grown in an atmosphere enriched in ^13^C (^13^CO_2_/CO_2_~11%), and was mixed with the flour obtained from soft wheat (*T. aestivum*, Crousty) grown in open fields. The ^13^C/^12^C of CHO in the cereal products (Table 1) was measured by gas chromatography/combustion/isotope ratio mass spectrometry (GC/C/IRMS) (see below).

### Experimental trials

The trials were conducted during the follicular phase of the menstrual cycle (6–10 days after the start of the menstrual period) and the breakfasts were ingested in a random balanced order. The subject reported to the laboratory at 0700 hours following a 10-h fast. For 3 days before each trial she also refrained from exercising, did not consume alcohol and ingested a diet poor in ^13^C providing 50–55% and 30–35% energy from CHO and fat, respectively, with the balance from protein. The subject was placed in a semi-supine position and venous catheters (Protect IV Plus 20G x 1″, Smith Medical, St Paul, MN, USA) were inserted in one forearm for administering [6,6-^2^H_2_]glucose and in the contralateral forearm for blood sampling.

**Figure 2:**
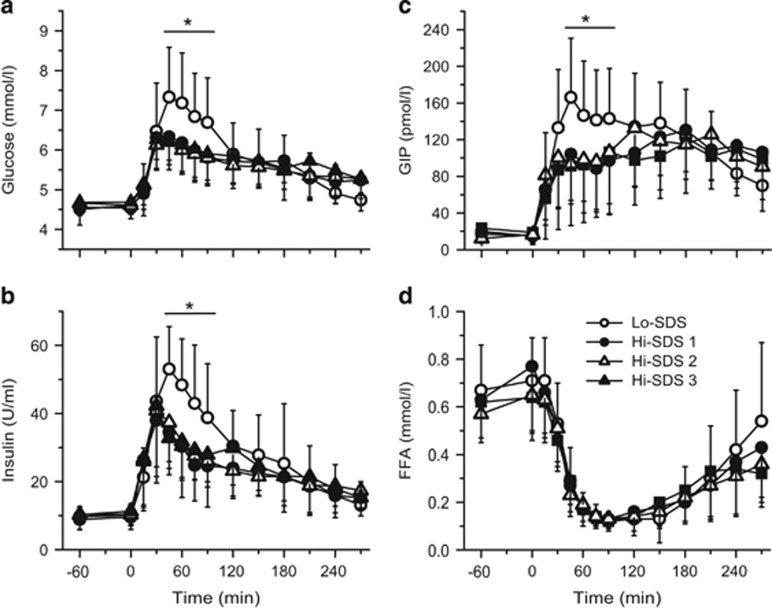
Plasma glucose (**a**), insulin (**b**), GIP (**c**) and FFA (**d**) concentrations following ingestion of breakfasts with a high or low content of SDS (Hi-and Lo-SDS): mean and s.d. (*n*=16 except for Hi-SDS 2, for which *n*=15); *P<0.05: significantly different from the Hi-SDS breakfasts.

Administration of [6,6-^2^H_2_]glucose (Eurisotop, Gif-sur-Yvette, France; bolus: 6.31±0.52 mg/kg; infusion: 0.062±0.012 mg/kg/min; mean±s.d.) was initiated 120 min before the breakfast (T-120) and pursued for 6.5 h (T270). The breakfast was ingested between T0 and T15. Arterialized blood samples (heated pad enveloping the hand and forearm) were withdrawn at 15- or 30-min intervals (Figure 2) in the preprandial and postprandial periods. Except for the measurement of plasma glucose, which was performed immediately (Glucose 2 Analyzer, Beckman Coulter, Fullerton, CA, USA), blood samples were transferred on gel tubes or tubes with EDTA (BD Vacutainer, VWR International, Radnor, PA, USA), blood cells were removed by centrifugation and the supernatant was distributed into cryotubes and stored at −80 °C until analysis for the measurement of ^2^H/H and ^13^C/^12^C in plasma glucose (see below) and of FFA (NEFA C, Wako Chemicals Gmbh, Neuss, Germany), insulin (Human insulin specific RIA kit, Linco Research, St Charles, MO, USA) and GIP concentrations (competitive radio-immunoassay: human GIP (Total) ELISA kit, Linco Research). The total volume of blood sampled was 150 ml for each trial.

### Glucose turnover

Isotopic composition of glucose was measured by gas chromatography/positive chemical ionization mass spectrometry (GC/EI-MS, GC 6890-MS 5973, Agilent Technologies, Massy, France) for ^2^H/H in plasma glucose and by GC/C/IRMS (Isoprime, Isoprime Ltd, Cheadle Hulme, UK) for ^13^C/^12^C in both glucose in the cereal products and in plasma glucose after derivatization in glucose penta-acetylaldononitrile.^18^ For this purpose, glucose from the cereal product was obtained by enzymatic hydrolysis^19^ and plasma glucose was purified by sequential anion–cation exchange chromatography (AG 50 W-X4 hydrogen and AG 1-X8 formate resins, Bio-Rad, Marnes-la-Coquette, France).^18^

Steele's equation for non-steady-state was used to compute RaT and RaE, as well as the rates of disappearance (RdT and RdE), from the percentage of [6,6-^2^H_2_]glucose^20^ and of ^13^C-glucose in plasma glucose^21^ as described previously.^22^ EGP was computed as RaT-RaE.

### Statistics

Data are reported as mean and s.d. Comparisons were made on a Gaussian repeated measurement model following examination of the normality of distribution (Shapiro–Wilk test, Henry line and skewness and kurtosis). If not verified as per the Shapiro–Wilk test, the parametric analysis was completed by nonparametric analysis with the analysis on the cumulative data from T0 to T270 (incremental or decremental area under the curve (iAUC and dAUC) or average values) representing the counterpart of the repeated measurement analyses. A two-sided level of 5% for the global type 1 risk was applied, the *P*-values being adjusted using the Sidak–Holm method for two-sided tests, for multiple comparisons. In the case of nonparametric tests, the type 1 risk was assessed with the Friedman test.

## Results

The small differences between the biscuits did not translate into significant difference between the three Hi-SDS breakfasts for any of the variables measured at any time point. These data have, thus, been pooled to compute the average values reported below.

### Plasma metabolite and hormone concentrations

When compared with the Lo-SDS breakfast, the glycemic response following the three Hi-SDS breakfasts was significantly lower between T45 and T90 (Figure 2): the peak value (at T45) and iAUC between T0 and T120 were, respectively, ~15% (*P*<0.001 for the three Hi-SDS breakfasts) and ~34% lower (*P*≤0.002). No significant difference was observed beyond T90. The decline in plasma glucose between T45 and T270 was three times faster following the Lo- than the Hi-SDS breakfasts (12 vs 4 μmol/l/min).

Differences in glycemic responses were associated with parallel differences in plasma insulin and GIP concentrations, which were both significantly lower with the Hi- than the Lo-SDS breakfast from T45 to T90 (Figure 2). Beyond T90, no significant difference between the four breakfasts was observed for insulin or GIP concentrations. When compared with the Lo-SDS breakfast, peak insulin values (at T30 and T45 with the Hi- and Lo-SDS breakfasts) were significantly lower by ~30–40% (*P*≤0.017 for the three breakfasts), and the iAUC between T0 and T120 was significantly lower by ~30–35% with the Hi-SDS breakfasts (*P*=0.002). Peak GIP concentration was also delayed and significantly lower with the Hi-SDS than with the Lo-SDS breakfast (Figure 2). Significant but moderate correlation coefficients were computed between plasma glucose, insulin and GIP concentrations (Table 2).

Plasma FFA concentration transiently but markedly fell in the postprandial period without any significant difference between the four breakfasts (Figure 2).

### Plasma glucose kinetics

**Figure 3:**
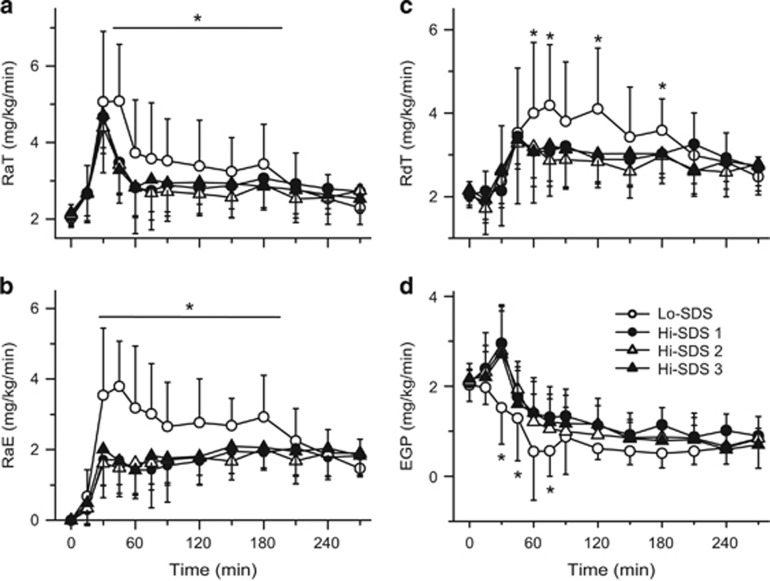
Rate of appearance of total and exogenous glucose (RaT and RaE): (**a**) and (**b**), rate of disappearance of total plasma glucose (RdT: (**c**) and rate of endogenous glucose production (EGP): (**d**) following ingestion of breakfasts with a high or low content of SDS (Hi-and Lo-SDS): mean and s.d. (*n*=16 except for Hi-SDS 2, for which *n*=15); *P<0.05: significantly different from the Hi-SDS breakfasts.

A large excursion of RaE was present with the Lo-SDS breakfast (Figure 3): following a rapid increase to the 3.79-mg/kg/min peak value at T45, RaE dropped to 2.65 mg/kg/ min at T90 and a further ~50% drop was observed between T180 and T270. In contrast, following the Hi-SDS breakfasts, RaE leveled off at ~1.8 mg/kg/min between T30 and T270. As a consequence, between T30 and T180, RaE was significantly lower following the Hi-SDS than the Lo-SDS breakfast (Figure 3; iAUC significantly lower by ~38–44%, *P*<0.001). No significant difference was observed between the breakfasts beyond T180.

When compared with the Lo-SDS breakfast, when the Hi-SDS breakfasts were ingested, peak RaT values were not significantly different (4.59 and 5.08 mg/kg/min, respectively, at T30), but RaT was significantly lower from T45 to 180 (Figure 3; iAUC ~30–45% lower between T0 and T270; *P*<0.001). When compared with RaT peak, RdT peak was delayed by 15 and 30 min with the Hi- and Lo-SDS breakfast, respectively (Figure 3), and the iAUC from T0 to T270 was significantly lower by ~30–50% following ingestion of the Hi- than the Lo-SDS breakfasts (*P*≤0.002).

The value of EGP computed using dual-tracer technique should be taken with caution.^23^ Indeed, the transient rise in EGP early in the absorptive period following the Hi-SDS breakfasts was an obvious artifact most probably due to uneven distribution of tracer and tracee in the various subpools of the glucose space^24, 25^ and/or that errors made in estimating RaT and RaE cumulate in the computation of EGP. Also, RaE represents the appearance in the peripheral circulation of (1) ^13^C-glucose absorbed from the gut and which escaped removal in the liver on first pass, (2) ^13^C-glucose removed by the liver early in the observation period and which could be released later, and (3) ^13^C-glucose synthesized in the liver from 3-carbon products^26, 27, 28, 29^ deriving from exogenous glucose in the gut or in peripheral tissues, and thus labeled with ^13^C. Consequently, EGP, computed as RaT-RaE, represents unlabeled glucose released from the liver, from unlabeled glycogen stores and gluconeogenesis from unlabeled precursors, and thus underestimates glucose release from the liver. However, beyond 60–90 min following CHO ingestion, the values of EGP computed using dual-tracer technique and the more accurate triple-tracer technique (with both one- and two-compartment models) are essentially similar.^23^ This suggests that when compared with the Lo-SDS breakfast, the lower decrease in EGP observed beyond T60 following ingestion of the Hi-SDS breakfasts (Figure 3) adequately reflects a smaller reduction in liver glucose output.

Between T0 and T180, the cumulative amount of glucose derived from ingested CHO was significantly lower by ~40% with the Hi- than with the Lo-SDS breakfast and the cumulative RaT was significantly lower by ~16% (*P*<0.001 for both variables) (Table 3). These differences disappeared between T180 and T270 but the total amount of exogenous glucose that appeared in the peripheral circulation over the entire postprandial period remained significantly lower by ~31% with the Hi- than with the Lo-SDS breakfasts (*P*<0.001).

## Discussion

As hypothesized, when compared with the Lo-SDS breakfast, following ingestion of the Hi-SDS breakfasts, the glycemic excursion was lower, with a much smaller increase between T0 and T120 and a slower return to pre-ingestion values. This was due to a much smaller excursion of RaE, which resulted in a lower challenge to plasma glucose homeostasis, that is, a more modest increase immediately after ingestion, a more stable value beyond T30 and a lower iAUC between T0 and T180. In addition, a smaller portion of the CHO ingested appeared in the peripheral circulation over the 270-min observation period following the Hi- than the Lo-SDS breakfasts, probably allowing for a larger sustained RaE and plasma glucose concentration at distance from the meal.

Also as hypothesized, the lower glycemic excursion following the Hi- than the Lo-SDS breakfast elicited a lower insulin response with a correlation computed in the interval T0–T120 between plasma glucose and insulin concentration, which was high and compared well with that reported by Eelderink *et al.*^12^ (0.79, *P*<0.01). The lower insulin response can also be partly due to the smaller increase in the concentration of GIP, which is partly responsible for the preabsorptive insulin response following a CHO meal.^30, 31^ The presence of glucose in the intestinal lumen is a potent stimulus for GIP release.^30, 31^ The lower GIP concentration with the Hi- than with the Lo-SDS breakfast, which is in line with data from Eelderink *et al.*,^12^ thus, is an evidence that glucose was released at a slower rate from the starch in the biscuits than in the extruded cereals. In further support to the hypothesis of cause and effect relationships between SDS content and (i) appearance of glucose in the intestinal lumen, (ii) stimulation of GIP release, (iii) glucose absorption, (iv) glycemic response and (v) stimulation of insulin secretion, moderate but significant correlations were found between T0 and T120, (i) between GIP concentration and RaE (the best surrogate for glucose absorption), (ii) RaE and glycemia, and (iii) GIP and insulin concentrations (Table 2) as also shown by Eelderink *et al.*^12^ (*r*=0.82, 0.63 and 0.60, all *P*<0.01).

These observations are in line with those from the four studies in which RaE has been compared following ingestion of similar amounts of starchy foods with well-characterized differences in their SDS content.^11, 12, 15, 32^ In these studies with various amounts of CHO ingested and large or narrow differences in SDS content between the two types of CHO tested, the iAUC of RaE was consistently 20–35% lower following ingestion of the starch with the higher SDS content, and this was associated with lower glycemic^15, 32^ and/or insulinemic responses^12, 15^ as well with a lower response of GIP.^12^ In a fifth study by Wachters–Hagedoorn,^33^ uncooked corn starch and corn pasta with a large difference in %SDS (45.3% and 6.8%, respectively) were ingested. No significant difference was observed in the AUC of RaE and the response of plasma glucose, insulin and GIP concentrations between the two meals. However, because of CHO loss in the cooking water, the amount of CHO available was much lower in the pasta than in the uncooked corn starch (50 vs 37.5 g), suggesting that for a similar amount of starch ingested, the higher the %SDS, the lower the response of RaE, and of plasma glucose, insulin and GIP concentration.

It is generally accepted that the glycemic response to starchy foods with a low GI is due to a slower rate of digestion of CHO resulting in a lower rate of glucose absorption and lower RaE and RaT.^10^ However, Schenk *et al.*^10^ have shown that the higher glycemic response observed with cornflakes compared with a bran cereal product was not due to a higher RaT, which was similar in the two products, but due to a transiently lower increase in RdT. In the study by Priebe *et al.*,^13^ when compared with glucose, the lower glycemic response observed with wholemeal wheat bread was not due to a lower RaE but due to a larger reduction in EGP. Finally, in the study by Eelderink *et al.*,^11, 12^ as already discussed, over the first 2 h following the meal RaE was ~32% lower with the pasta (10.6% SDS vs 5.4% SDS in the bread) but the glycemic responses were similar. This was not due to a lower reduction in EGP, but due to a lower clearance rate of plasma glucose (which reflects RdT).

In the present experiment, unlike in these studies, EGP, RaT and RdT, and the glycemic response to the two types of breakfasts paralleled the response of RaE. The magnitude of all of these responses was lower following ingestion of the Hi- than the Lo-SDS breakfasts. The recruitment of the control mechanisms responsible for restricting the excursion of the glycemic response in the postprandial period was, thus, precisely matched to the disturbance in plasma glucose homeostasis due to the surge in RaE. This match can be tracked back to the lower insulin response to the Hi- compared with the Lo-SDS breakfast, which in turn was probably due to the lower response of plasma glucose and GIP concentration.

From a practical point of view, these observations confirm that substituting extruded cereals with one of the three plain biscuits at breakfast will slow down glucose appearance in the peripheral circulation, reduce the challenge to plasma glucose that follows the meal and the associated excursions in plasma glucose and insulin concentrations, and distribute the glucose ingested over a longer period. This might improve plasma glucose control and provide long-term health benefits in subjects with glucose intolerance^34^ and also in the general population.^4, 5^

## Figures and Tables

**Table 1:** Composition of the cereal products and breakfasts

	*Hi-SDS breakfasts*			*Lo-SDS breakfast*
	*Biscuit 1*	*Biscuit 2*	*Biscuit 3*	*Extruded cereals*
*Starch*				
Total (g)	33.0	36.9	36.9	34.1
SDS (g)	15.3	17.0	15.5	0.1
RDS (g)	18.9	21.0	24.5	36.4
SDS/RDS	0.81	0.81	0.63	<0.01
SDS/total	0.46	0.46	0.42	<0.01

*Cereal product*				
Mass (g)	72	72	72	66
Energy (kJ)	1381 (330 kcal)	1373 (328 kcal)	1377 (329 kcal)	1213 (290 kcal)
Protein (g)	4.5	5.0	5.0	4.6
Fat (g)	11.9	11.3	11.4	7.3
Fibers (g)	1.9	2.1	2.2	2.1
Available CHO (g)	51.6	51.6	51.8	51.5
Sugars (g)	18.5	14.6	14.6	17.4
Sugars/available CHO	0.36	0.28	0.28	0.34
GI	46	50	52	60
Insulin index	63	50	57	63
^13^C/^12^C (δ^13^C ‰/VPDB)	−10.2	−11.0	−11.0	−12.4

*Skimmed milk*				
Volume (ml)	150	150	150	150
Energy (kJ)	209 (50 kcal)	209 (50 kcal)	209 (50 kcal)	209 (50 kcal)
Protein (g)	5	5	5	5
Fat (g)	0.3	0.3	0.3	0.3
Sugars (g)	6.9	6.9	6.9	6.9

*Vegetable oil*^a^				
Mass (g)	—	—	—	4.5
Energy (kJ)	—	—	—	171 (41 kcal)

*Total*				
Energy (kJ)	1590 (380 kcal)	1582 (378 kcal)	1586 (379 kcal)	1593 (381 kcal)
Protein (g)	9.5	10.0	10.0	9.6
Fat (g)	12.2	11.6	11.7	12.1
CHO (g)	58.5	58.5	58.7	58.4
Coffe or tea (ml)^b^	300	300	300	300

Abbreviations: CHO, carbohydrate; GI, glycemic index; RDS, rapidly digestible starch; SDS, slowly digestible starch; VPDB, Vienna Pee Dee Belemnitella standard (^13^C/^12^C = 0.0112372).

a Vegetable oil (2.25 g rapeseed oil and 2.25 g palm oil) was added to the Lo-SDS breakfast for compensating for the lower content of fat in the extruded cerals than in biscuits.

b Unsweetned and decaffeinated or detheinated.

**Table 2:** Correlation coefficients between glucose, insulin and GIP concentrations, and RaE following the breakfasts

	*Glucose*	*Insulin*	*GIP*
Insulin	0.744 (1)		
GIP		0.493 (1)	
		0.632 (2)	
RaE			0.600 (1)
			0.624 (2)

Between T0 and T120 (1) and T0 and T270 (2). Significantly different from zero, *P*<0.001.

**Table 3:** Cumulative appearance, in grams, of total and exogenous glucose, and EGP over selected time intervals during the post-prandial period in the four trials (mean±standard deviation)

	*Hi-SDS breakfasts*			*Lo-SDS breakfast*
	*Biscuit 1 (*n=*16)*	*Biscuit 2 (*n=*15)*	*Biscuit 3 (*n=*16)*	*Extruded cereals (*n=*16)*
*T0–T180*				
Total	31.9±4.0	30.7±4.8	33.0±6.0	37.8a±8.4
Exogenous	16.0±4.4	16.2±3.6	17.9±5.6	28.1a±7.2

*T180–T270*				
Total	15.1±2.8	14.1±1.6	14.3±2.4	14.4±3.2
Exogenous	10.0±3.2	9.9±2.4	10.5±2.8	11.0±3.2

*T0–T270*				
Total	47.1±6.8	44.8±6.0	47.4±7.2	52.2a±9.2
Exogenous	26.0±5.2	26.1±5.2	28.4±7.6	39.1a±8.0
EGP	21.1±4.4	18.6±3.6	18.9±4.0	13.0a±2.8

Abbreviations: EGP, endogenous glucose production; SDS, slowly digestible starch.

a Significantly different from the three Hi-SDS breakfasts, *P*<0.05; differences were assessed using one-way analysis of variance for repeated measurements and the Sidak–Holm *post hoc* test.

## Notes

AM and SV are employees of the Nutrition Department, Mondelēz International R&D; FP is an occasional consultant for the Nutrition Department, Mondelēz International R&D; RRL and FP received an unrestricted grant from Mondelēz International R&D to conduct this work. The remaining authors declare no conflict of interest.
